# Comparison of maximal lactate steady state with anaerobic threshold determined by various methods based on graded exercise test with 3-minute stages in elite cyclists

**DOI:** 10.1186/s13102-020-00219-3

**Published:** 2020-11-17

**Authors:** Kamila Płoszczyca, Dominik Jazic, Zofia Piotrowicz, Małgorzata Chalimoniuk, Józef Langfort, Miłosz Czuba

**Affiliations:** 1 Department of Kinesiology, Institute of Sport, Trylogii 2/16, 01-982 Warsaw, Poland; 2 Department of Sports Theory, The Jerzy Kukuczka Academy of Physical Education, Katowice, Poland; 3 Faculty of Health Sciences, Jan Dlugosz University, Czestochowa, Poland

**Keywords:** Maximal lactate steady state, Anaerobic threshold, Endurance performance, Exercise testing, Cycling, Blood lactate

## Abstract

**Background:**

The maximal lactate steady state (MLSS) is defined as the highest workload that can be maintained for a longer period of time without continued blood lactate (LA) accumulation. MLSS is one of the physiological indicators of aerobic performance. However, determination of MLSS requires the performance of a series of constant-intensity tests during multiple laboratory visits. Therefore, attempts are made to determine MLSS indirectly by means of anaerobic threshold (AT) evaluated during a single graded exercise test (GXT) until volitional exhaustion. The aim of our study was to verify whether AT determined by maximal deviation (D_max_), modified maximal deviation (ModD_max_), baseline LA concentration + 1 mmol/l (+ 1 mmol/l), individual anaerobic threshold (IAT), onset of blood lactate accumulation (OBLA_4mmol/l_) and V-slope methods based on GXT with 3-min stages provide valid estimates of MLSS in elite cyclists.

**Methods:**

Twelve elite male cyclists (71.3 ± 3.6 ml/kg/min) completed GXT (the increase by 40 W every 3 min) to establish the AT (by D_max_, ModD_max_, + 1 mmol/l, IAT, OBLA_4mmol/l_ and V-slope methods). Next, a series of 30-min constant-load tests to determine MLSS was performed. Agreement between the MLSS and workload (WR) at AT was evaluated using the Bland–Altman method.

**Results:**

The analysis revealed a very high (r_s_ > 0.90, *p* < 0.001) correlation between WR_MLSS_ and WR_Dmax_ and WR_IAT_. The other AT methods were highly (r_s_ > 0.70) correlated with MLSS except for OBLA_4mmol/l_ (r_s_ = 0.67). The Bland-Altman analysis revealed the highest agreement with MLSS for the D_max_, IAT and + 1 mmol/l methods. Mean difference between WR_MLSS_ and WR_Dmax_, WR_IAT_ and WR_+1mmol/l_ was 1.7 ± 3.9 W, 4.3 ± 7.9 W and 6.7 ± 17.2 W, respectively. Furthermore, the WR_Dmax_ and WR_IAT_ had the lowest limits of agreement with the WR_MLSS._ The ModD_max_ and OBLA_4mmol/l_ methods overestimated MLSS by 31.7 ± 18.5 W and 43.3 ± 17.8 W, respectively. The V-slope method underestimated MLSS by 36.2 ± 10.9 W.

**Conclusions:**

The AT determined by D_max_ and IAT methods based on the cycling GXT with 3-min stages provides a high agreement with the MLSS in elite cyclists. Despite the high correlation with MLSS and low mean difference, the AT determined by + 1 mmol/l method may highly overestimate or underestimate MLSS in individual subjects. The individual MLSS cannot be properly estimated by V-slope, ModD_max_ and OBLA_4mmol/l_ methods.

## Background

Anaerobic threshold (AT) is a load above which internal homeostasis is suddenly disturbed and fatigue is intensified, accompanied by changes in gas exchange and blood lactate (LA) concentration during exercise [1, 2]. This point is one of the most significant physiological variables in endurance sports. Anaerobic threshold (AT) has been used to diagnose the sports performance of athletes and to determine exercise zones used during training. The AT shift towards higher loads is considered to be an indicator of improved performance in endurance sports [2, 3]. Anaerobic threshold (AT) is much more reflective of training-induced changes in aerobic performance than changes maximal oxygen uptake (VO_2max_) [3] and can be improved with appropriately selected training loads and training methods, even in well-trained athletes [4–7]. It should be noted, however, that AT can change depending on diets [8–11] and supplementation [12–14], and may be modified by environmental conditions, such as altitude or temperature [15, 16], and psychological factors [17, 18]. Paradoxically, higher AT can be observed in overtraining due to changes in LA blood levels as a response to exercise [19]. Therefore, test results should be interpreted with caution, taking into account the presence of numerous factors that may affect AT.

Over several decades, many concepts of AT determination have been developed, which are based on the observation of the increase in blood LA levels and/or changes in respiratory indices recorded during graded exercise tests (GXTs) [2]. Another approach to evaluate aerobic performance is to determine maximal lactate steady state (MLSS). MLSS is defined as the highest workload that can be maintained for a longer period of time without continued blood LA accumulation. During the exercise at and below the MLSS workload, a balance occurs between LA production and its rate of clearance. When the effort is above the MLSS workload, the rate of LA production exceeds its removal [20]. By definition, MLSS is attained when blood LA levels increases by less than 1 mmol/l between 10 and 30 min of the constant-intensity exercise test [21].

Determination of MLSS requires the performance of a series of constant-intensity tests during multiple laboratory visits, which in practice is burdensome and may disrupt the athletes’ training program. Therefore, attempts are made to determine MLSS indirectly based on AT evaluated during a single GXT until volitional exhaustion [22–28]. However, the results comparing various concepts of AT with MLSS are conflicting [22–28].

It seems that some of the discrepancies in the literature concerning the application of individual methods of evaluation of AT to determine MLSS result from differences in GXT protocol design (step duration and load) and different types of test exercise (cycling, running, rowing). The choice of the appropriate load and time between increments during GXT is critical for the evaluation of AT based on LA levels [22, 27, 29, 30]. It is suggested that in order to achieve lactate steady state during GXT and valid determinations of the lactate threshold (LT), step duration should be longer than 6 min [22, 30–32]. This is related to the time needed to transport LA from the intramuscular compartment to plasma, which depends on the capacity of the monocarboxylic transporters (MCT) [33]. With too short a step duration, a blood LA level can result from lower load compared to the one at which it was recorded, which can lead to AT overestimation.

In training practice, not only LT, but also VO_2max_ and maximal heart rate (HR_max_) are usually determined during GXT. The extension of the time of stages results in a significant increase in the time of the entire test, which may result in VO_2max_ and HR_max_ not being achieved due to premature local muscle fatigue [30, 34, 35]. Therefore, a 3 to 4-min stages are very often used during GXT [36–39]. For this reason, it is important to determine which AT method yields the most favorable estimation of the MLSS with such a step duration.

In addition to GXT study design and exercise mode, the sports performance level of the athlete can play an important role in determining AT and its agreement with MLSS. Training adaptations lead to changes in the metabolic response to exercise loads and increased exercise tolerance [40]. The method that reflects MLSS well in people with lower levels of sports performance will not necessarily provide a favorable MLSS estimation in elite athletes.

Therefore, the aim of our study was to verify whether AT determined by D_max_, ModD_max_, + 1 mmol/l, IAT, OBLA_4mmol/l_ and V-slope methods based on GXT with 3-min stages provide valid estimates of the MLSS in elite cyclists. We hypothesize that using the GXT with AT determination allows for the indirect determination of MLSS in elite cyclists.

## Methods

### Participants

Fourteen male elite cyclists were recruited for this study. Two participants withdrew from participation because of infections. Twelve athletes completed all the testing and were included for analysis (aged 25.9 ± 3.2 years; body height 181.7 ± 4.4 cm; body mass 72.3 ± 5.3 kg; fat content (%) 8.6 ± 2.1%; VO_2max_ 71.3 ± 3.5 ml/kg^/^min). All cyclists had at least 6 years of national and international competition experience and were familiar with our laboratory testing procedures. All athletes had current medical examinations, without any contraindications to performing exhaustive exercise in a hypoxic environment. The participants provided their written voluntary informed consent before the participation. The research project was conducted according to the Helsinki Declaration and was approved by the Ethics Committee for Scientific Research at the Jerzy Kukuczka Academy of Physical Education in Katowice, Poland.

### Experimental design

The research was conducted at the end of the preparatory period. Testing procedures were identical for each athlete. The experiment was divided into two series of tests performed in a laboratory environment. All participants were familiarized with the test protocols before the first evaluations. The athletes were instructed to avoid strenuous exercise and caffeine intake for 24 h prior to each test. Throughout the experiment (from the 2 days prior to the experiment to the last MLSS test), participants consumed meals that contained the same amount of kcal, protein, fats, and carbohydrates each day (40 kcal/kg of body weight, 50% carbohydrates, 20% proteins, 30% fats). Participants stayed at the camp and consumed meals served only on the spot which were prepared according to the dietitian’s recommendations.

### Experimental testing

In the first series of testing, before breakfast, body mass and body composition were evaluated using the electrical impedance technique (Inbody 220, Biospace Co., Japan). Next, 2 h after a light breakfast (5 kcal/1 kg of body weight, 50% carbohydrates, 20% proteins, 30% fats), the GXT was performed to determine metabolic thresholds and aerobic capacity with an Excalibur Sport ergometer (Lode BV, Netherlands). The GXT started at a workload of 40 W, which was increased by 40 W every 3 min until volitional exhaustion. During the test, all cyclists were instructed to remain in a sitting position and maintain a cadence of 80 rpm (±5 rpm). Each cyclist’s bike setup (saddle height, reach, handle bar height) was recorded and reproduced for all tests.

During the GXT, heart rate (HR), oxygen uptake (VO_2_), expired carbon dioxide (CO_2_) and minute ventilation (VE) were measured continuously with a gas analyzer MetaLyzer 3B-R2 (Cortex, Germany) using the breath-by-breath method. The criterion of reaching VO_2max_ was respiratory exchange ratio (RER) above 1.1. The maximal workload (WR_max_) was indicated as the last completed stage of the progressive test. If a participant terminated the test before completing a given workload, the WR_max_ was calculated from the formula WR_max_ = WR_k_ + (t/T x WR_p_), where WR_k_ is the previous workload, t is exercise duration with the workload until premature failure, T is the duration of each workload, and WR_p_ is the amount of workload by which exercise intensity increased during the test [41]. Fingertip capillary blood samples for the assessment of LA levels (Biosen C line Clinic, EKF-diagnostic GmbH, Germany) were drawn at rest and at the end of each step of the test, as well as during the 3rd, 6th, 9th, and 12th minute of recovery.

The anaerobic threshold (AT) was determined using the D_max_ method [42], modified D_max_ method – ModD_max_ [36], the IAT method [43], + 1 mmol/l method [44], OBLA_4mmol/l_ method [45], and the non-invasive V-slope method [46]. V-slope workload was identified in the exercise intensity which, in a plot of the minute production of CO_2_ over the minute utilization of oxygen (VO_2_), shows an increase in the slope of above 1.0 [46]. Two independent investigators detected ventilator thresholds following the criteria previously described. If they did not agree, the opinion of a third investigator was sought.

In the second series of the tests, which started after a day of active recovery, all participants started to perform a series of efforts at a constant load to determine the MLSS. Each test was preceded by a 10-min warm-up with an individually set load of 65–70% HR_max_ and a fixed cadence of 80 rpm. The warm-up was followed by an increase in the load to the target value at which the athlete performed exercise for 30 min. The participants started a series of tests with an individual load equal to the lowest value of the threshold load determined by selected methods during the GXT performed in the first series of tests. Capillary blood samples were obtained from the fingertip at rest, at the end of the warm-up and after every 5 min of the test (5, 10, 15, 20, 25 and 30 min) to determine the LA concentration in the blood. When the LA concentration during the last 20 min of the test was stable and did not increase by more than 1 mmol/l, the test was repeated after a day of active rest with a load increased by 20 W. Tests with constant intensity were performed until a load was reached, during which the LA gain in blood exceeded 1 mmol/l in the last 20 min, and the previous load was considered as MLSS. Furthermore, if during the first test at a threshold load the LA increase exceeded the target value, the test was analogically repeated at a load lower by 20 W until the MLSS was reached. The use of our previous experience in indirect evaluation of MLSS [23] resulted in the determination of MLSS for the 2nd or 3rd time.

### Statistical analysis

The normality of the distribution of variables was checked using the Shapiro-Wilk test. A Wilcoxon test was used to assess significant differences between the MLSS and the AT determined by various methods. Agreement between the MLSS and workload at AT was evaluated using the Bland–Altman method [47]. The correlations were calculated using Spearman’s rank correlation coefficient. The statistical significance was set at *p* < 0.05. Statistical analyses were conducted using StatSoft Statistica 13.0 software.

## Results

### Maximal lactate steady state

The mean value of workload in MLSS (WR_MLSS_) was 298 ± 21 W. The mean values of VO_2_, HR and LA at the 10th and 30th min of the MLSS test are reported in Table 1.

**Table 1:** The mean values of VO_2_, HR and LA at the 10th and 30th min of MLSS test

Variables	MLSS test	
	10 min (Mean ± SD)	30 min (Mean ± SD)
VO_2_ (ml/kg/min)	58.5 ± 2.9	62.0 ± 3.4
HR (bpm)	174 ± 5.4	181 ± 6.2
LA (mmol/l)	3.67 ± 0.59	4.51 ± 0.61

*VO*_*2*_ oxygen uptake, *HR* heart rate, *LA* blood lactate concentration

### Graded exercise test

The mean values of WR_max_ and VO_2max_ were 409 ± 34 W and 71.3 ± 3.6 ml/kg/min, respectively. The HR_max_ and VE_max_ were 196 ± 5 bpm and 188.4 ± 18.4 l/min, respectively. The blood LA level increased by 7.42 ± 1.23 mmol/l during the GXT and it decreased by 2.46 ± 0.75 mmol/l during 12 min of the recovery period after the exercise.

### The MLSS and the various AT concepts

**Fig. 1:**
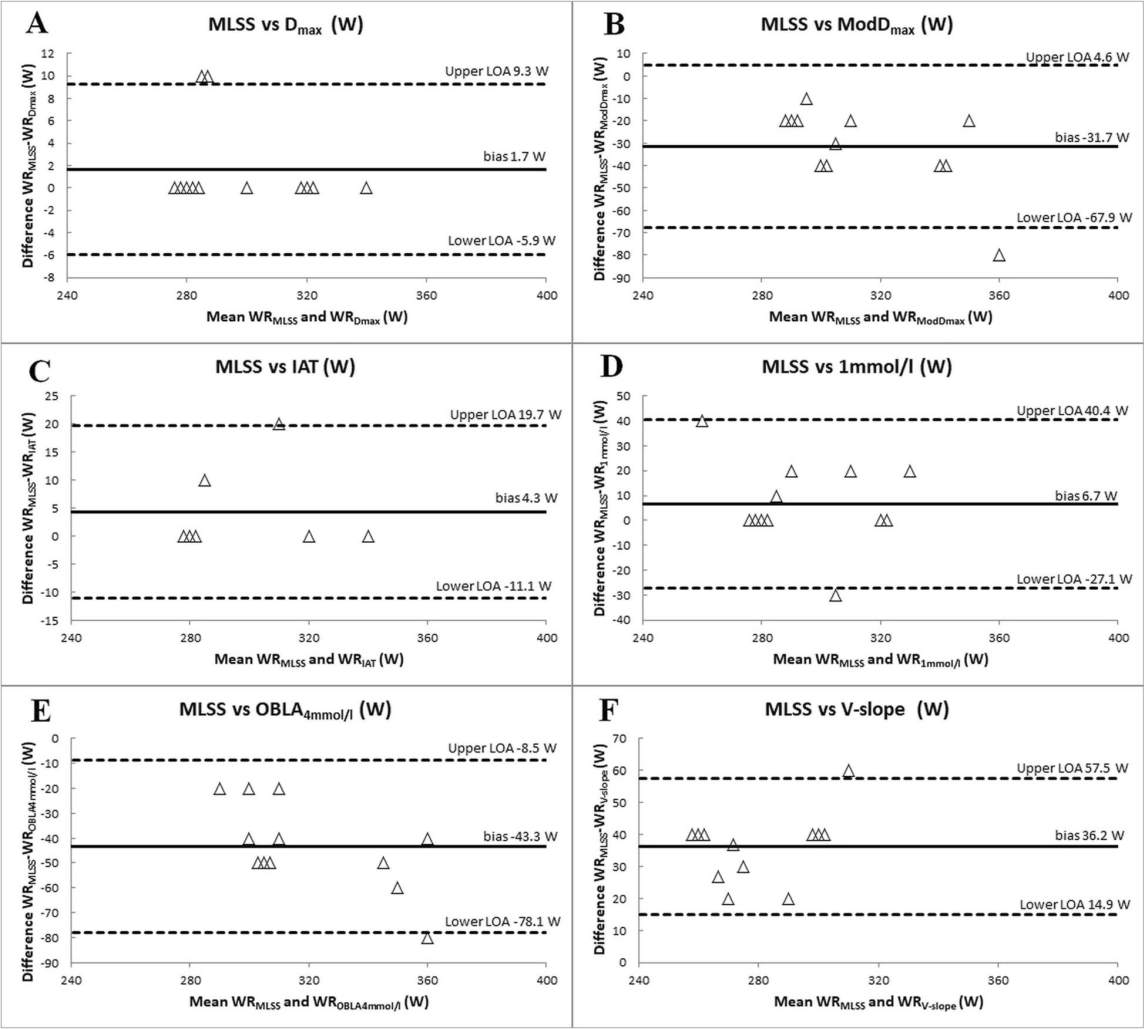
Bland-Altman plots comparing MLSS and workload at AT determined by D_max_ (**a**), ModD_max_ (**b**), IAT (**c**), + 1 mmol/l (**d**), OBLA_4mmol/l_, (**e**), V-slope (**f**) methods. The horizontal solid line represents the mean difference between the two measures (bias). The horizontal dashed lines represent the limits of agreement (LOA). *n* = 7 for IAT method and *n* = 12 for D_max_, ModD_max_, + 1 mmol/l, OBLA_4mmol_/l and V-slope

Comparisons of the WR_MLSS_ and the workload at AT determined by the six methods were presented in Table 2. The Wilcoxon test revealed significant differences (*p* < 0.01) between WR_MLSS_ and WR_VAT_, WR_ModDmax_ and WR_OBLA4mmol/l_. There were no significant differences between WR_MLSS_ and WR_Dmax_, WR_+1mmol/l_ and WR_IAT_. The analysis revealed a very high (r_s_ > 0.90, *p* < 0.001) correlation between WR_MLSS_ and WR_Dmax_ and WR_IAT_. Other AT methods were highly (r_s_ > 0.70) correlated with MLSS except for OBLA_4mmol/l_ (r_s_ = 0.67). The Bland-Altman analysis revealed the highest agreement with MLSS (i.e., low mean difference) for the D_max_, IAT and + 1 mmol/l methods. The mean difference between WR_MLSS_ and WR_Dmax_, WR_IAT_ and WR_+ 1mmol/l_ was 1.7 ± 3.9 W, 4.3 ± 7.9 W and 6.7 ± 17.2 W, respectively. Furthermore, the WR_Dmax_ and WR_IAT_ had the lowest limits of agreement with the WR_MLSS_ (Table 2). The limits of agreement for + 1 mmol/l method were large (upper LOA of 40.4 W and lower LOA of 27.1 W). The WR_V-slope_, WR_ModDmax_, WR_OBLA4mmol/l_ showed low agreement with MLSS (high mean difference and high limits of agreement). The ModD_max_ and OBLA_4mmol/l_ methods overestimated MLSS by 31.7 ± 18.5 W and 43.3 ± 17.8 W, respectively. The V-slope method underestimated MLSS by 36.2 ± 10.9 W. The relation between the workload at AT determined by various methods and MLSS was presented in the Bland-Altman plots (Fig. 1).

**Table 2:** Differences between the MLSS and the workload according to various AT concepts

	Mean ± SD (W)	Me (W)	Wilcoxon test p	MD (W)	Me_diff_ (W)	Upper LOA (W)	Lower LOA (W)	r_s_
**MLSS**	298.3 ± 21.2	290						
**D**_**max**_	296.7 ± 22.3	280	0.180	1.7	0.0	9.3	−5.9	0.93
**ModD**_**max**_	330.0 ± 32.5	320	< 0.01	−31.7	−25.0	4.6	−67.9	0.80
**IAT**	297.1 ± 24.3	280	0.180	4.3	0.0	19.7	−11.1	0.93
**+ 1 mmol/l**	291.7 ± 24.8	280	0.249	6.7	0.0	40.4	−27.1	0.79
**OBLA** **4 mmol/l**	341.7 ± 32.1	330	< 0.01	−43.3	−45.0	−8.5	− 78.1	0.67
**V-slope**	262.2 ± 17.2	260	< 0.01	36.2	40.0	57.5	14.9	0.89

*SD* standard deviation, *Me* median, *p* significance of differences, *MD* mean difference, *LOA* 95% limits of agreement, *Me*_*diff*_ median of differences, *r*_*s*_ Spearman’s rank correlation coefficient

## Discussion

The results of our study indicate that of the six methods included in our analysis, D_max_ and IAT methods yielded the most favorable estimations of the MLSS. The WR_Dmax_ and WR_IAT_ had the highest correlation (r_s_ > 0.90) and the lowest mean difference with the WR_MLSS_. The D_max_ method only slightly underestimates MLSS (by 1.7 ± 3.9 W) and 95% of differences between measurements by the MLSS and D_max_ method range from − 5.9 to 9.3 W. Likewise, the IAT slightly underestimates MLSS (by 4.3 ± 7.9 W), with limits of agreement between − 11.1 and 19.7 W. It should be noted that in our study, IAT was only evaluated for seven cyclists because of the rapid decline of the post-exercise LA value. This is a commonly reported issue with this method [24, 48]. For this reason, despite the favorable MLSS estimation, the IAT method may not be useful in practice for some athletes, especially those highly-trained.

Despite the lack of significant differences (*p* > 0.05) and low bias (MD = 6.7 ± 17.2 W) between WR_MLSS_ and WR_+ 1mmol/l_, the limits of agreement for comparison between these variables suggest that the extent of disagreements is too high to allow MLSS to be accurately estimated using + 1 mmol/l method in individual participants. The V-slope, ModD_max_ and OBLA_4mmol/l_ methods failed to provide valid estimates of the MLSS. We observed large mean differences (− 43 to 36 W) between AT determined by these methods and MLSS.

In our study, AT determined by the D_max_ method based on the cycling GXT test with 3-min stages (GXT3) showed a high agreement with the MLSS. The results of previous research in this area are divergent. Arratibel-Imaz et al. [49] demonstrated a high agreement of D_max_ with MLSS (MD = -2.1 W, *r* = 0.93) using cycling GXT3 in cyclists and triathletes. Similarly, Czuba et al. [23] showed a high correlation (*r* = 0.97) between WR_Dmax_ (GXT3) and WR_MLSS_ in well-trained female and male cyclists. Results obtained by Pallarés et al. [25] revealed less bias (− 1.8 W) but with lower correlation (*r* = 0.56) between D_max_ (GXT1) and MLSS in well-trained cyclists. On the contrary, Van Schuylenbergh et al. [22] showed that despite a correlation between D_max_ (GXT6) and MLSS (*r* = 0.85), WR_Dmax_ was lower by 22 W compared to WR_MLSS_ in elite cyclists. Similar findings were reported by Jamnick et al. [27] for four GXTs with different stage durations (3, 4, 7, and 10 min). The authors observed a high correlation between D_max_ and the MLSS (*r* = 0.94 to 0.97) but mean differences were too large (19 to 49 W) for D_max_ to provide a valid estimate of the MLSS. However, it is worth noting that in the research carried out by Jamnick et al. [27], WR_Dmax_ determined during GXT3 showed the smallest mean difference compared to MLSS. As the duration of the stage increased (from 3 to 10 min), the difference between WR_MLSS_ and WR_Dmax_ increased. The discrepancy of the above results may result from different test protocols and different sports performance of the study participants. However, with the D_max_ method used in highly-trained cyclists, GXT with 3-min stages seems to be best suited for the indirect MLSS determination by the D_max_ method. Extending the stage to 6–10 min increases the MLSS underestimation by D_max_ [22, 27].

Our results indicate that IAT methods (GXT3), similarly to D_max_, yielded high agreement with MLSS. To the best of our knowledge, this is the first study to analyze whether the IAT method provides valid estimates of the MLSS in elite cyclists. Previous research carried out using the cycling exercise test, De Barros et al. [48] revealed good agreement between IAT (GXT3) and MLSS (*r* = 0.85, MD = -0.7) in active males. The difference between MLSS and IAT was lower than 25 W for all subjects. Furthermore, Hauser et al. [24] indicated that IAT (GXT4) was highly correlated with MLSS (*r* = 0.83) and overestimated it by 12 W. However, the large individual differences were reported in this study, which was likely to result from heterogenous endurance level of study participants.

It should be noted that a major practical limitation of the IAT method is that the determination of AT depends on post-exercise changes in blood LA level. For this reason, the determination of AT is infeasible in athletes with a fast rate of LA utilization during restitution. Such a problem may occur more often in highly-trained endurance athletes. This is due to the increased efficiency of MCT, increased capillarity and a more efficient use of LA in metabolic processes by tissues with high oxidative potential [50–52]. For this reason, although MLSS can be properly estimated by IAT, the use of this method in the sports practice may be difficult.

The third method which in our study showed small mean differences with MLSS is the + 1 mmol/l method (MD = 6.7). However, focusing on the individual determination of MLSS, it should be concluded that + 1 mmol/l (GXT3) is not a valid method for MLSS estimation in elite cyclists. The lack of agreement of + 1 mmol/l with MLSS in cyclists was also previously reported by Pallarés et al. [25] but they used a different exercise protocol (ramp test; GXT1, with capillary blood sampling obtained every 2 min).

The large individual variation in the agreement of WR_+ 1mmol/l_ and WR_MLSS_ can be attributed to an arbitrarily imposed LA increase by 1 mmol/l above baseline [44]. The resting LA level and the rate of increase of LA level in the blood during exercise show great interindividual variation and depend on the sports performance [53, 54], diet, and supplementation [13, 55, 56], and may be modified by environmental conditions [15, 16] and psychological factors [17, 18]. Therefore, the determination of a specific absolute value of the LA level or its increment (as in the + 1 mmol/l, ModD_max_, and OBLA methods) may result in the erroneous determination of method-specific points and the consequent overestimation or underestimation of AT and also MLSS. Furthermore, it should be noted that accurate determination of baseline LA concentration is needed for the + 1 mmol/l method to be reliable. This may prove problematic due to the fluctuations associated with analyzer error [57], which later affects the determination of AT. For the above reasons, attempts to use the + 1 mmol/l method for the determination of MLSS in individual athletes should be made with care, taking into account factors that may interfere with its repeatability and reliability.

The results of our study indicate that ModD_max_, OBLA_4mmol/l,_ and V-slope are not valid methods for estimation of MLSS in cyclists when GXT with 3 min stage duration is used. We showed that ModD_max_ and OBLA_4mmol/l_ overestimate, and the V-slope method underestimates MLSS exercise intensity by more than 30 W, with large individual deviations. Such a large discrepancy is unacceptable in elite cyclists, who need a high degree of precision in controlling and monitoring the training process. This is especially true when this process is regulated by recording the power generated by the cyclist during the exercise.

In contrast to the presented findings, a recent study carried out by Jamnick et al. [27] and Zwingmann et al. [28] showed that the ModD_max_ method is a good MLSS determinant for cyclists and triathletes. However, their studies involved athletes with a lower sports performance than those examined in our study (MLSS: 264 W and 229 W vs. 298 W), which may have led to the discrepancies in the obtained results.

In the ModD_max_ method, an important role in determining AT is played by the first rise in blood lactate concentration of > 0.4 mmol/l. It should be taken into account that the fixed value of 0.4 mmol/l does not take into account the individual level and kinetics of LA. It can be presumed that the moment of this increase depends on stage duration, load, and sports performance of the athlete. A longer stage and a higher increase in the load result in an increase in LA at earlier stages. Furthermore, our many years of unpublished observations show that the increase in sports performance is associated with a clear flattening of the lactate curve at sub-threshold loads. This is mainly due to an increase in the consumption of fatty acids by muscles (shifting the point of intersection of carbohydrate and fat utilization towards higher loads), and an improvement in the utilization of LA [51, 58]. This phenomenon causes that a line formed by the point preceding the first rise in blood LA concentration of > 0.4 mmol/l and the final LA point has a steep profile in highly-trained athletes. Consequently, LT determined by the ModD_max_ method in highly trained athletes occurs at higher loads than LT determined by D_max_ and IAT methods. For this reason, MLSS can also be greatly overestimated by ModD_max_, as we observed in our study. Given the above, we suggest that the ModD_max_ method cannot be used to indirectly determine MLSS in elite cyclists. ModD_max_ is likely to be useful in people with lower levels of sports performance [26, 28], but this aspect requires further research.

The results obtained in this study indicate that OBLA_4mmol/l_ (GXT3) does not provide a correct estimation of MLSS. This result is in line with several previous studies using GXT cycling with a stage length of 1–10 min. It was observed that OBLA_4mmol/l_ significantly overestimates [25, 27] or underestimates MLSS [48] and shows a great individual variation [24]. The main reason for these discrepancies is that the OBLA_4mmol/l_ method is based on the association of threshold load values with a specific constant blood LA level, without taking into account individual variability and dynamics of changes in circulating LA concentration (LA curve) during exercise [2]. The assumption of the OBLA_4mmol/l_ method was to select blood LA concentration which is similar to MLSS. The vast majority of subjects achieve MLSS for LA of ca. Four millimoles per Liter, but this concentration occurs after a much longer time than the 3 min adopted in GXT3, which is related to the previously described LA outflow into the blood. Furthermore, individual variability in blood LA levels at MLSS has also been demonstrated [24, 59], as observed in our study, despite the homogeneous sports performance level of the cyclists studied. The final blood LA concentration during MLSS test ranged from 3.6 to 5.6 mmol/l. Therefore, arbitrary setting the LA level at 4 mmol/l especially during GXT3 will result in the low agreement of WR_OBLA4mmol/l_ with WR_MLSS_. It is suggested that extending the stage duration to 7–10 min while decreasing the set LA level to 3–3.5 mmol/l will increase OBLA agreement with MLSS [27]. However, this does not eliminate the interindividual variability in blood LA level, which in our opinion excludes the use of the OBLA method in indirect determination of MLSS in elite cyclists.

The V-slope method is based on a visual evaluation of the curve of the relationship between VO_2_ and VCO_2_. V-slope analysis detects the onset of excess CO_2_ production occurring in response to an increase in hydrogen ion (H^+^) concentration during exercise [46]. To minimize changes in blood pH, H^+^ ions are buffered by the bicarbonate buffer (HCO_3_^−^), which leads to the formation of carbonic acid that dissociates to H_2_O and CO_2_. Excess CO_2_ stimulates ventilation, whereas higher ventilation results in increased CO_2_ release [60].

The V-slope method is used in practice as a non-invasive method of AT determination. However, VAT and LT do not always occur at the same workload [60]. Some studies indicate the unreliability of the V-slope method both as an alternative to LT [42, 61, 62] and the MLSS in cyclists [25]. In our study, we demonstrated that AT determined using the V-slope method is achieved at lower loads than MLSS (MD = 36 W), which is consistent with the results obtained by Pallarés et al. [25]. The discrepancies between WR_V-slope_ and WR_MLSS_ are due to the fact that athletes improve their MCT system as a result of endurance training and increasing the intramuscular LA utilization. This adaptive mechanism does not directly affect the H+ ion concentration and thus the CO_2_ production [63]. The above mechanism explains the shift (towards lower loads) of the V-slope threshold (based on the increase in CO_2_) in relation to LT and MLSS (based on LA changes in blood).

## Conclusion

The AT determined by D_max_ method based on cycling GXT with 3-min stages provides high agreement with the MLSS in elite cyclists. A similar agreement with MLSS is ensured by IAT, however, in some individuals, the determination of AT using this method is unworkable due to the rapid decline of LA after exercise. Therefore, the use of the IAT method to estimate MLSS in the sports practice may be difficult. Despite the high correlation with MLSS and low mean difference, the AT determined by + 1 mmol/l method may highly overestimate or underestimate MLSS in individual subjects. The individual MLSS cannot be properly estimated by V-slope, ModD_max,_ and OBLA_4mmo/l_ methods.

## Data Availability

The datasets used and/or analysed during the current study are available from the corresponding author on reasonable request.

## Abbreviations

MLSS: Maximal lactate steady state
AT: Anaerobic threshold
GXT: Graded exercise test
WR: Workload
D_max_: Lactate threshold determined by D_max_ method
ModD_max_: Lactate threshold determined by modified D_max_ method
IAT: Individual anaerobic threshold
+ 1 mmol/l: Lactate threshold determined by + 1 mmol/l method
OBLA_4mmo/l_: 4 mmol/l lactate threshold
V-slope: Ventilatory threshold determined by V-slope method
